# Supplementary material for: DMRforPairs: identifying Differentially Methylated Regions between unique samples using array based methylation profiles
Source: BMC Bioinformatics. 2014 May 15;15:141. doi: 10.1186/1471-2105-15-141 (PMC4046028; doi:10.1186/1471-2105-15-141)
Supplement: Additional file 2 — DMRforPairs output for the comparison of A431-MCF7 and NA17018-NA17105. Please start from the HTML files in each folder. Available via the BMC Bioinformatics website. [file 1471-2105-15-141-S2.zip › 1394847754114233_MOESM2_ESM/A431_MCF7/figures/30953.pdf]

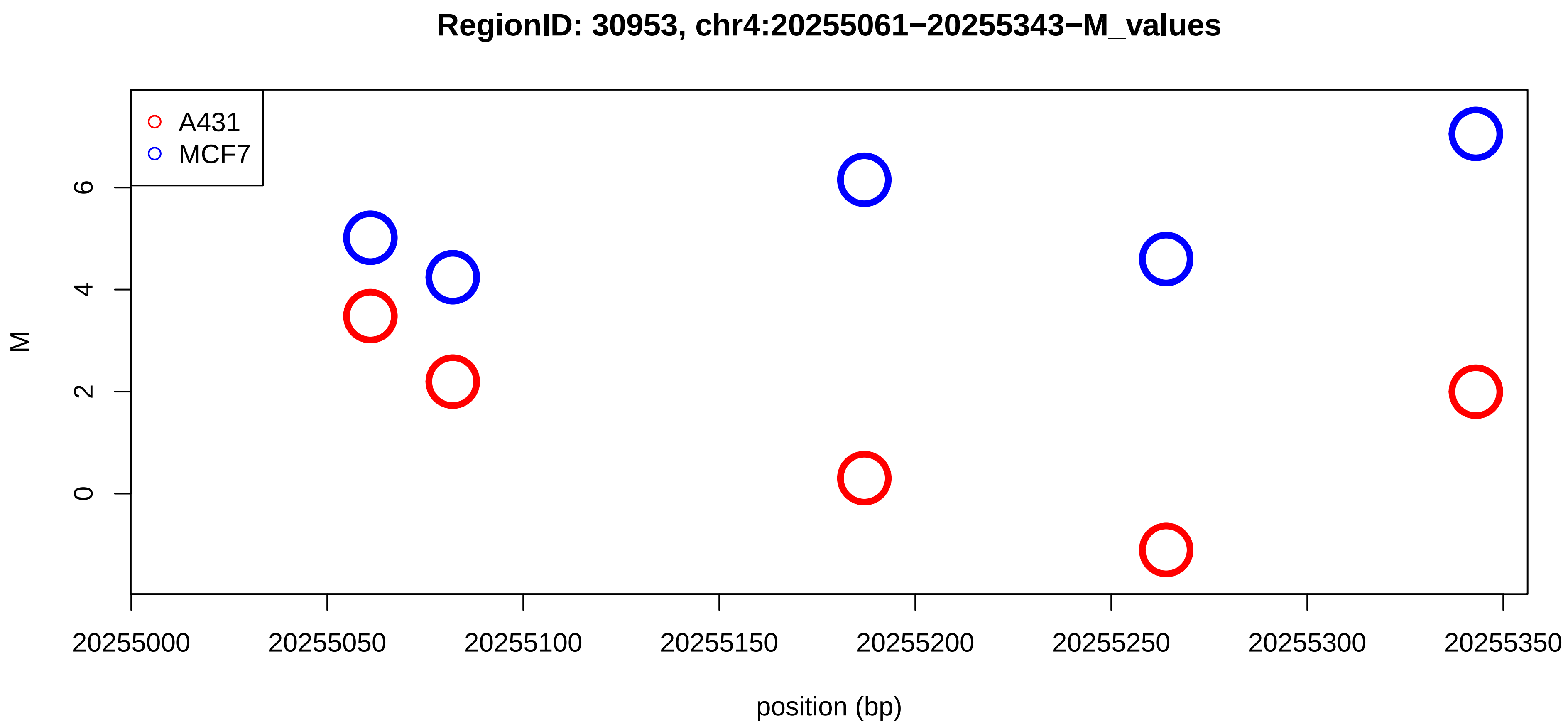

RegionID: 30953, chr4:20255061−20255343−M_values
A431
MCF7
M
0
2
4
6
20255000
20255050
20255100
20255150
20255200
20255250
20255300
20255350
position (bp)

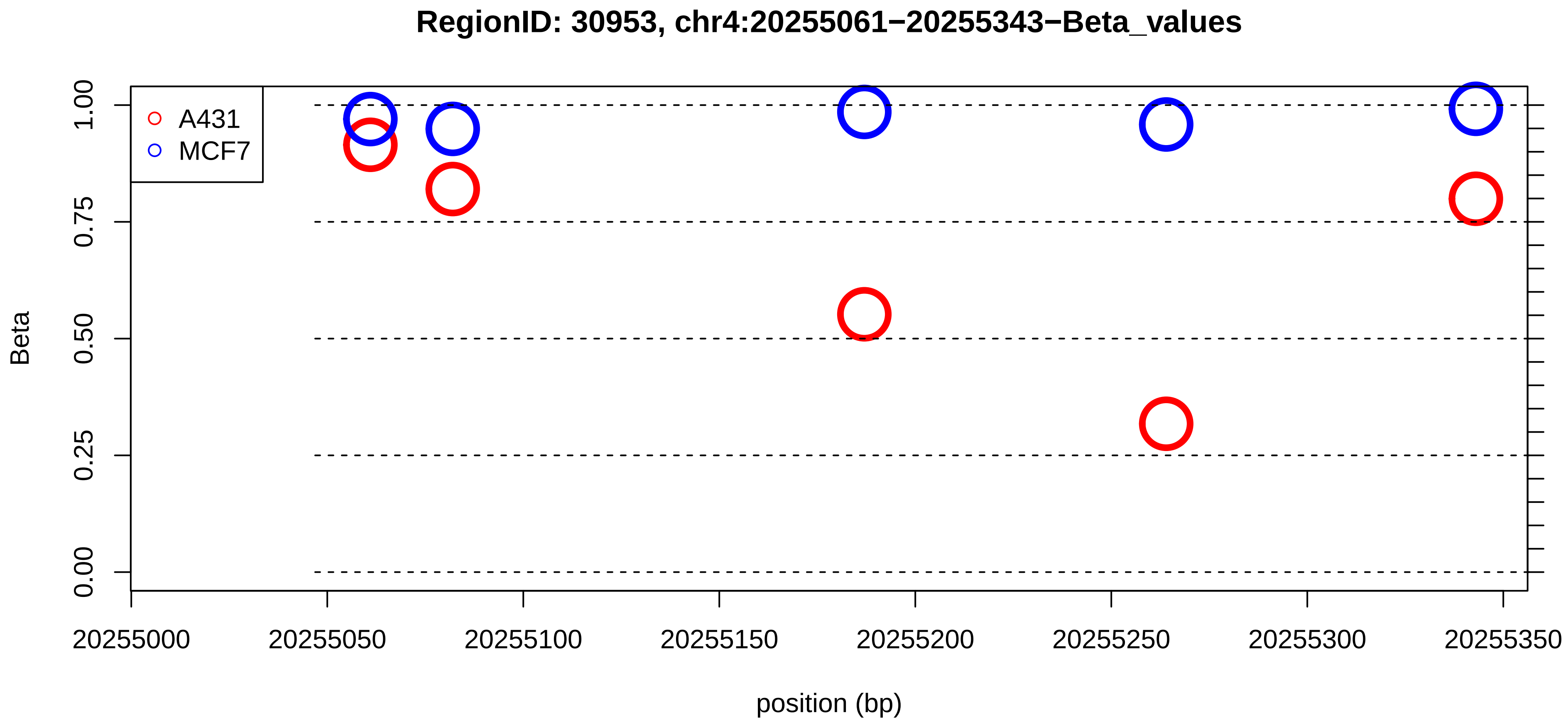

RegionID: 30953, chr4:20255061−20255343−Beta_values
A431
MCF7
Beta
0.00
0.25
0.50
0.75
1.00
20255000
20255050
20255100
20255150
20255200
20255250
20255300
20255350
position (bp)

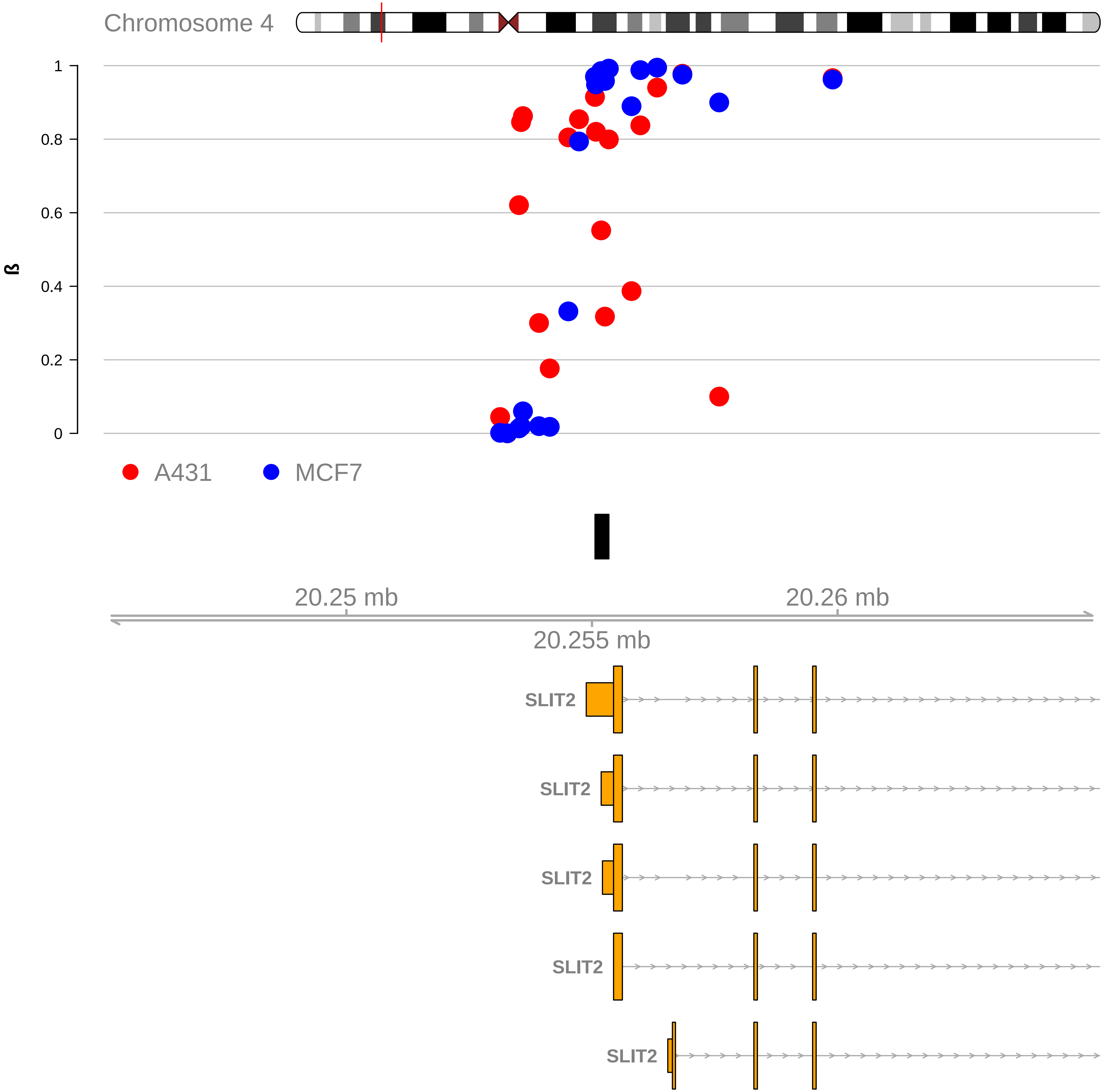

Chromosome 4
1
0.8
0.6
0.4
0.2
0
ß
A431
MCF7
20.25 mb
20.26 mb
20.255 mb
SLIT2
SLIT2
SLIT2
SLIT2
SLIT2
